# Effect of Primary and Secondary Beads of Carbon Enterosorbent on Haematological Parameters and Oxidative Stress Development Caused by Melphalan in Rats

**DOI:** 10.3390/medicina55090557

**Published:** 2019-09-02

**Authors:** Oksana Shevchuk, Elisaveta Snezhkova, Veronika Sarnatskaya, Victor Mikhailenko, Alexei Glavin, Lyudmyla Makovetska, Kvitoslava Bardakhivska, Inna Birchenko, Oleksandr Kozynchenko, Volodymyr Nikolaev

**Affiliations:** 1 I. Horbachevsky Ternopil National Medical University, 46001 Ternopil, Ukraine; 2 R.E. Kavetsky Institute of Experimental Pathology, Oncology and Radiobiology (IEPOR) of the National Academy of Science of Ukraine, 03022 Kyiv, Ukraine; 3 ImmutriX Therapeutics Inc., Rapid City, SD 57703, USA

**Keywords:** oxidative stress, myelotoxicity, anti-cancer chemotherapy, rats, melphalan, enterosorption

## Abstract

*Background and Objectives:* Side effects of anti-cancer drugs are usually accompanied by oxidative stress, including myelotoxicity. We evaluated the potential of oral highly activated micro-/macroporous carbon adsorbents (bulk density of 0.16 g/cm^3^, surface area calculation by Brunauer–Emmett–Teller model (S_BET_) > 2200 m^2^/g, derived from proprietary phenolic resin beads) to alleviate oxidative stress and myelotoxicity in rats. *Materials and Methods:* A single injection of cytostatic melphalan (L-PAM) at a dose of 4 mg/kg was used for modelling. Two forms of activated carbon were used: AC1—primary beads with the particle size range of 125–250 µm, and AC2—micronized AC1 with a mean particle size of ~1 µm. We measured haematological parameters white blood cells, red blood cells, platelet count, and haemoglobin level. Oxidative stress intensity was evaluated using the following markers: total levels of reactive oxygen species (ROS) in blood plasma; catalase activity (CAT) and pro-oxidant/antioxidant ratio in blood haemolysate samples; level of reduced glutathione (GSH) in liver tissues; oxidative modification of proteins, OPM (APHD, aldehyde–dinitrophenylhydrazone derivatives and KPHD, ketone dinitrophenylhydrazone derivatives) and malonic dialdehyde (MDA) in blood plasma and liver samples. *Results:* AC2 administration promoted significant myeloprotective effect: 1.5-fold increase in leukocytes, 2-fold in neutrophils, 1.5-fold in lymphocytes, and 1.23-fold in platelet count compared to the experimental Melphalan Group. At the same time, AC1 administration resulted in a slight increase in haematological parameters. Both ACs positively corrected important, but diverse, components of oxidative stress. They significantly reduced oxidative modification of blood and liver proteins (especially the AC1 form), normalized the level of reduced glutathione, pro-oxidant/antioxidant ratio and other markers. For some markers, such as ROS production in blood plasma, the use of enterosorbents resulted in non-significant a shift towards normal parameters. *Conclusions:* Oral activated carbon adsorbents reduce oxidative stress intensity and myelotoxicity; they can be promising means to combat the adverse effects of chemotherapy in clinical practice.

## 1. Introduction

Cancer is among the top causes of mortality in the world with between 15 and 70% of all deaths depending on the development of public health system in different countries according to World Health Organization data. It has a significant economic and social impact [1]. Today, the combined use of surgery, radiation therapy and anti-cancer chemotherapy results in improved outcomes for people with malignant tumours. However, antineoplastic drugs have severe side effects, such as bone marrow toxicity (myelosuppression), damage to gastrointestinal epithelium (mucositis), impaired wound healing, loss of hair, sterility, teratogenicity, carcinogenicity, etc. [2,3,4,5,6,7]. Among the most serious side effects is bone marrow suppression and its consequences: anaemia, thrombocytopenia, and neutropenia, which may lead to the delay and interruption of chemotherapy treatment and reduction of its efficacy. Intestine epithelium, a fast-changing tissue, is another vulnerable site. Its damage results in mucositis, significantly decreasing the quality of life of patients and limiting their ability to tolerate optimal tumoricidal treatment [6,7,8]. Oxidative stress plays an essential role in the pathogenesis of adverse effects of anti-cancer chemotherapy [9,10,11]. Developing and implementing new approaches to reduce side effects of antitumor treatment (such as via modulation of excess oxidation processes) is an important objective of modern medicine.

Activated carbon materials are widely used in medicine: for blood purification (haemoperfusion) by direct perfusion of blood through an adsorbent column; for cleansing of contaminated wounds to promote healing (application sorption therapy) and as oral adsorbents for enteral sorption therapy (enterosorption) [12,13,14]. They were shown to be excellent adsorbents for gastrointestinal decontamination to remove endo- and exotoxins from the body. Sorption detoxification also demonstrated promising results in reducing nausea and vomiting in oncology patients, as well as improving outcomes in the renal and hepatic failure of various origins, in purulent surgery and others [13,15,16,17,18]. State-of-the-art nanotechnological methods are utilised to tailor porosity of carbon adsorbents of different origin and adjust their adsorptive properties to meet specific goals [19].

Our previous studies of melphalan-induced bone marrow suppression demonstrated significant myeloprotective activity of oral granulated activated carbon adsorbent with bulk density γ = 0.18 g/cm^3^ and granule diameter of 0.15–0.25 mm [20]. This is an improved version of highly activated synthetic SCN carbons (bulk density of 0.3–0.4 g/cm^3^) used for reducing cyclophosphane toxicity in the 1980-s [21]. Another oral adsorbent Carboline (highly activated fibrous carbon material) was effective in decreasing systemic toxicity of the cytotoxic agent cisplatin [22]. This study focuses on myeloprotective properties of activated carbons as expressed by improved signs and consequences of excess lipid and protein peroxidation, processes that are involved in the pathogenesis of common side effects of tumoricidal treatment.

An aggravating negative impact of cancer therapy is the increase in nausea and vomiting, severe mucositis and stomatitis, which are very painful and uncomfortable for the patients. Such factors significantly decrease the quality of life and ability to eat causing a range of negative emotions [6,7]. Orally disintegrating micronized forms (secondary orally-dispersed granules) of solid drugs improve their palatability and are more comfortable to consume under chemotherapy-induced nausea and vomiting [23,24,25]. Clinical potential of orally disintegrating micronized forms in comparison to traditional pharmaceutical forms of the same activated carbon oral adsorbent (primary solid beads) thus becomes an important study point. 

The purpose of the study to evaluate the effects of two forms of ImmutriX carbon enterosorbents, derived from the same highly activated phenolic carbon with optimized porous structure, on the haematological parameters and oxidative stress markers after a single injection of alkylating agent melphalan in rats.

## 2. Materials and Methods

### 2.1. Oral Adsorbents Preparation and Characteristics

**Figure 1:**
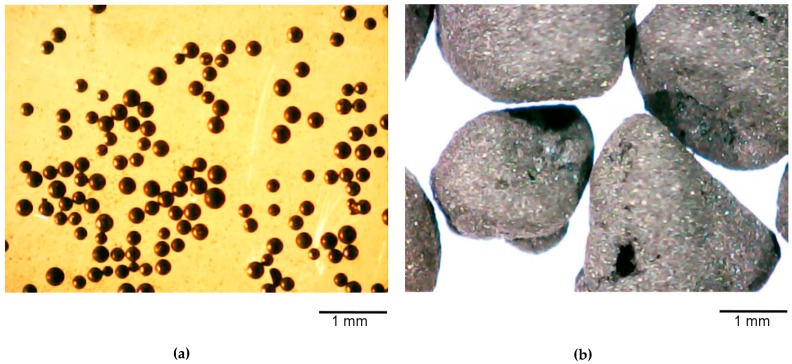
(**a**) Primary beads, activated carbon 1 (AC1), ×20, (**b**) Activated carbon 2 (AC2) secondary beads bound in granules, ×20.

We used two highly activated phenolic carbon [26] forms as adsorbents. The first form, AC1, consists of primary solid beads of activated carbon with a diameter 0.15–0.25 mm. These are produced by pyrolysis of proprietary porous phenolic resin and further activation of the carbonized material in carbon dioxide flow at 1050 °C (Figure 1a). The second form, AC2, is a secondary orally disintegrating formulation consisting of the micronized AC1 with the diameter of microparticles about 1 µm and 5% of starch as a binder (Figure 1b).

**Figure 2:**
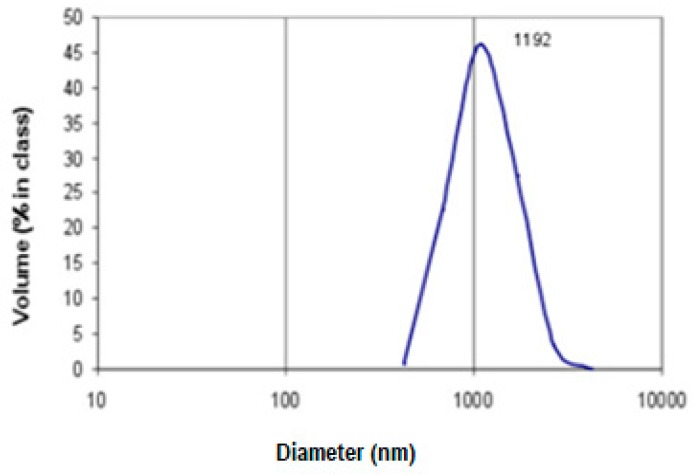
The size distribution of carbon microparticles component of AC2.

Activated carbon beads AC1 were micronized using an automatic multichannel milling system MKM-300H (Millicom Ltd, Kharkiv, Ukraine). To evaluate particle size distribution after micronization, the sample was suspended in pure water (200 µg mL^−1^) and the size distribution was examined in 1L aliquots by dynamic light scattering using a Malvern Instruments Zetasizer (Worcestershire, UK) equipped with He-Ne laser (25 mW, λ = 633 nm) with an angle detection of 90° at 22 °C. The results are presented in Figure 2.

**Figure 3:**
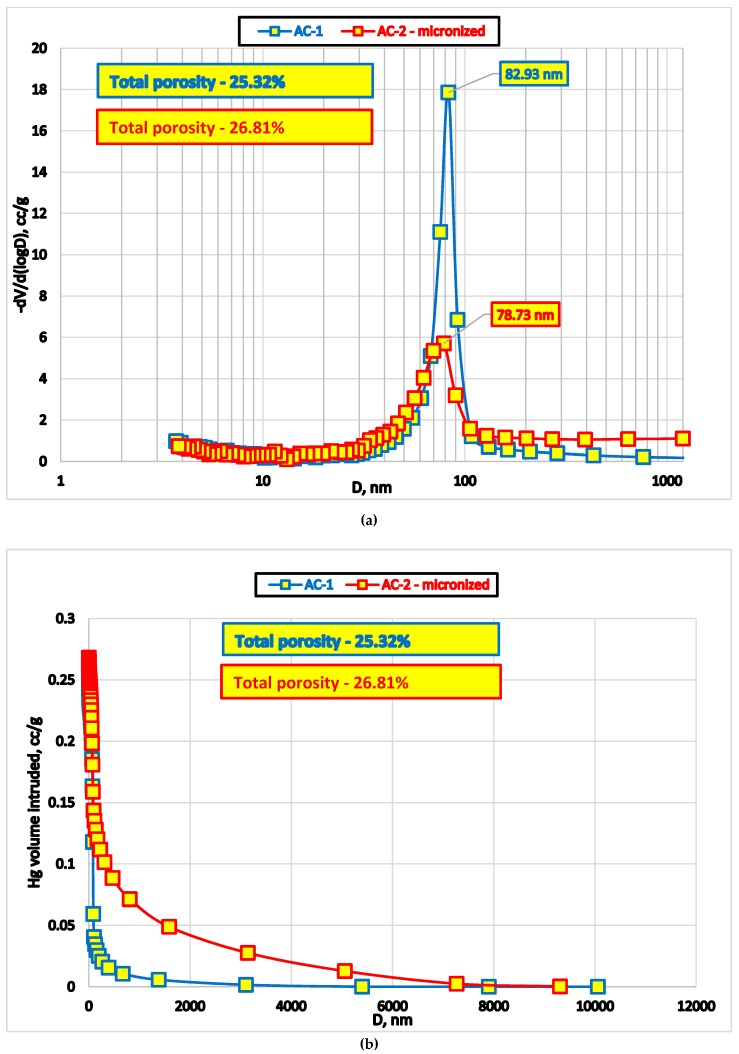
Pore size distribution of AC1 (primary beads) and AC2 (secondary micronized beads) measured by mercury porosimetry: (**a**) log pore diameter (nm), (**b**) pore diameter (nm).

We used standard methods to evaluate surface area and pore size distribution: low-temperature nitrogen adsorption Micromeritics TriStar II Plus (Micromeritics Instrument Co., Norcross, GA, USA) and mercury intrusion porosimetry in high-pressure mode (Poremaster, Quantachrome Instruments, New York, NY, USA). Data acquisition was performed using respective instruments’ software (Figure 3).

Results of mercury porosimetry demonstrate that micro-grinding of AC1 oral adsorbent destroys certain proportion of macropores (approximately 80 nm, Figure 3).

Additional mercury intrusion at the lower end of the pressure range into ‘pseudopores’ at the tail of the main peak (Figure 3b) is likely a result of the voids surrounded by microparticles of different size.

**Figure 4:**
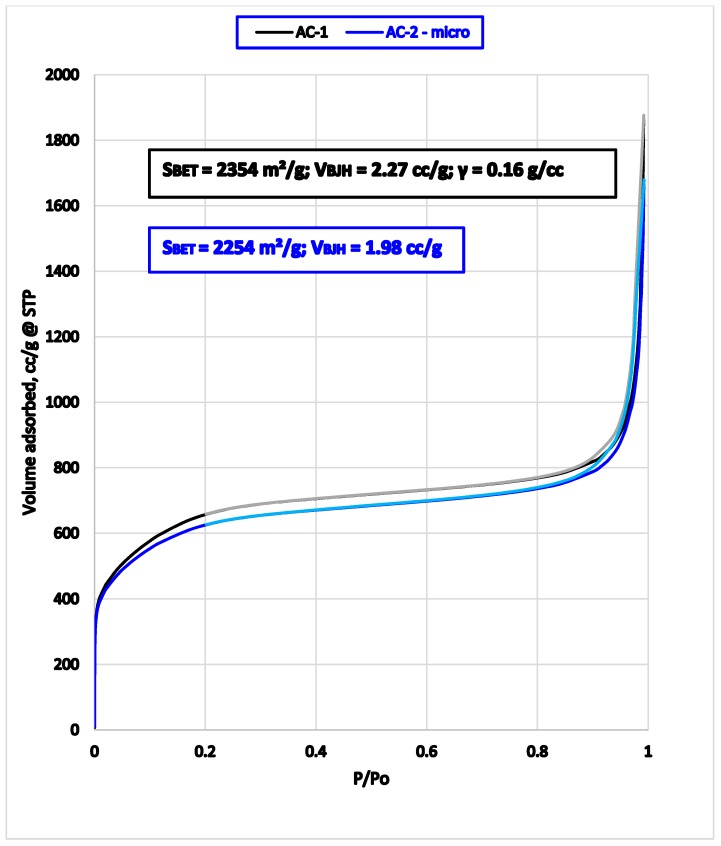
Absorption and desorption curves for AC1 and AC2 sorbents. Notes: Lighter colours of the curves show desorption, darker—adsorption process. S_BET_—the surface area calculated from BET (Brunauer-Emmett-Teller) method.

Low temperature nitrogen adsorption and desorption curves for AC1 and AC2 sorbents point to substantial similarity in porosity parameters (Figure 4). Shallow hysteresis curves between adsorption and desorption branches indicate perfect interconnectivity of the macropores.

For Methylene Blue (MB) adsorption evaluation, 12 mL of MB solution (1.5 mg/mL in phosphate buffered saline (PBS)) was added to 100 mg of the adsorbent and concentration of MB was assessed at set time intervals by direct absorption spectrophotometry at 620 nm (Bio-Tek Instruments, Inc., VT, USA). 

The kinetic data of Methylene Blue adsorption (C_16_H_18_ClN_3_S × 3 H_2_O, M.W. = 373.9 D) from its aqueous solution are presented in Table 1.

Data in Table 1 indicate that, despite well-developed and interconnected transport porosity of activated carbon AC1 primary beads, it took more than 1 day to saturate their adsorption sites to arrive at a relatively constant value of dye concentration. In contrast, microparticles in the secondary granules required shorter time to reach the equilibrium point. This variation between the kinetic qualities of primary beads and micronized particles suggests a marked difference in their biological and therapeutic properties. To test this proposition and to determine the most effective form of the enterosorbent derived from the same activated carbon precursor, we performed an experimental animal study using alkylating agent Melphalan.

### 2.2. Design of the Experiment

The experiment was performed on white inbred female albino rats, of 220 ± 20 g primary weight. The rats were reared at the R.E. Kavetsky Institute of Experimental Pathology, Oncology and Radiobiology (IEPOR) animal facility (Kyiv, Ukraine). A normal light-dark cycle was maintained for the animals; they were fed typical rodent chow diet with tap water ad libitum.

These experiments were performed in IEPOR accordingly the contract № V-11-17 dated 24 February 2017. The experimental work with animals was previously approved by the protocol № 1a dated 16 February 2017 on the meeting of Bioethics Commission of IEPOR.

In this experiment, the animals (*n* = 35) were randomly assigned to four groups: Group 1—control group (*n* = 5); Group 2 (*n* = 10)—rats that received Melphalan intravenously (Melphalan Group); Group 3 (*n* = 10) received both Melphalan and enterosorbent AC1 (Melphalan + AC1 Group); and Group 4 (*n* = 10)—rats that received both Melphalan and AC2 enterosorbent (Melphalan + AC2 Group).

Melphalan (GlaxoSmithKline Manufacturing S.p.A., Parma, Italy and Glaxo Operations UK Limited, Barnard Castle, UK) was injected in the tail vein at a single dose of 4.0 mg/kg. Rats of the Control Group received injections of saline in the equivalent dose.

The dose of carbon primary beads and secondary granules was 10 mL per 1 kg of rat body weight. The administered dose of carbon beads was mixed with a small amount of oatmeal as a binder. It was fed to the rats 3 h after and 3 h before a regular meal once each day starting 2 days before and ending 7 days after the Melphalan injection. Group 1 (control) and Group 2 rats received oatmeal balls without adsorbent. The animals were kept in separate cages for the visual control of oatmeal consumption.

The rats were weighed and then sacrificed using general ether anaesthesia on the 8th day after Melphalan injection. We harvested blood plasma and haemolysates, as well as liver, thymus, spleen, small intestine, gonadal tissue samples for further studies.

### 2.3. Evaluation of Haematological Parameters

Automated XS Hematology Analyzer (Sysmex Corporation, Kobe, Japan) was used to determine blood cell counts: white blood cell count (WBC), red blood cell count (RBC), haemoglobin level (Hb), platelet count (Pt), neutrophil and lymphocyte counts.

### 2.4. Evaluation of Oxidative Stress Markers

We used the following parameters to evaluate the extent of oxidative stress: reactive oxygen species (ROS) levels in blood plasma; catalase activity (CAT) and pro-oxidant/antioxidant ratio in blood haemolysate samples; reduced glutathione (GSH) levels in liver tissues; oxidative modification of proteins, OPM (APHD, aldehyde-dinitrophenylhydrazone derivatives and KPHD, ketone dinitrophenylhydrazone derivatives) and malonic dialdehyde (MDA) in blood plasma and liver samples. All reagents were purchased from Sigma–Aldrich (St. Louis, MO, USA).

Blood samples were stored in a refrigerator at 3–5 °C prior to obtaining plasma and lymphocytes. To prepare haemolysate, the blood was diluted 800 times with distilled water and stored at the same temperature. Blood plasma was separated by centrifugation for 15 min at 3000 rpm and room temperature. Part of the blood plasma was frozen and stored at −70 °C. To study ROS levels, 400 μL of the blood was centrifuged for 30 seconds at 10,000 g, plasma samples were immediately frozen and stored in liquid nitrogen. Liver samples were collected at 3–5 °C and subsequently stored in liquid nitrogen. Liver homogenates were prepared by grinding the liver tissue in 0.05 M Tris–HCl buffer (pH 7.4) in the proportion of 1 g of liver per 5 mL of buffer. The procedure was carried out at 3–5 °C. Protein concentration in blood plasma and liver homogenates was determined following Greenberg and Craddock (1982) [27].

#### 2.4.1. Assessment of ROS Levels in Blood Plasma

The total amount of ROS in blood plasma was determined using the *N*,*N*-dimethyl-p-phenylenediamine (DPD) method [28]. This colorimetric method is based on the oxidation of DPD to coloured radical cations in the presence of oxygen radicals and Fe^2+^, forming a double peak in the absorption spectrum (λ_abs_ = 511 and 552 nm). The measurements were performed using an automatic Synergy microplate reader (BioTek Instruments, Inc., Winooski, VT, USA), Five μL of blood serum was placed into each clear-bottomed well, 145 μL of 0.1 M acetate buffer (pH 4.8) was added and the sample was incubated for 3 min at 37 °C. After that, 100 μL of DPD and ferric sulphate in acetate buffer (with final concentration of 100 μg/mL and 4.7 μM, total volume in the well of 250 μL) was added, and the absorbance at 511 nm was recorded every 30 s for 6 min from the start of the reaction. The total amount of ROS was calculated using a calibration curve with standard H_2_O_2_ solutions (0, 0.37, 0.74, 1.47, 2.21, 2.94, 3.68 and 4.41 mM) and expressed in mM H_2_O_2_ per 1 L of plasma per min (mM/L/min). The data in 1–4 min interval, when a linear formation of the coloured DPD form was observed, were used for calculations.

#### 2.4.2. Assessment of Reduced Glutathione Concentration 

Reduced glutathione (GSH) levels were determined following Baker, Cerniglia, and Zaman (1990) [29], and the results were expressed in nM per mg protein (nM/mg). In addition, 800 μL of 20% trichloroacetic acid was added to 400 μL of liver homogenate in Tris buffer and incubated at 5–10 °C for 30 min. Supernatant was obtained by centrifugation at 6000 g for 15 min at 5 °C. Distilled water was added to the control sample instead of the homogenate. Each test tube contained 15 μL of the supernatant, 225 μL of 0.3 M disodium hydrophosphate (pH = 7.7) and 38 μL of 0.4% of 5,5′-dithio-bis-(2-nitrobenzoic acid) (DTNB). After incubating for 20 min following the addition of DTNB, absorbance of the samples at a wavelength of 412 nm was measured at room temperature.

#### 2.4.3. Measurement of Catalase Activity

The activity of antioxidant enzyme catalase (CAT) was determined in the blood haemolysate of the experimental rats using Koroliuk et al. (1988) methodology [30], which is based on the ability of hydrogen peroxide to form a stable coloured complex with molybdenum salts. The method was modified for use with a plate reader. The reaction was carried out in test tubes containing 0.8 mL of 20 mM hydrogen peroxide and 40 mL of the sample (blood diluted with distilled water 800 times). After 10 min, the reaction was stopped by adding 0.4 mL of 4% ammonium molybdate ((NH_4_)_2_MoO_4_) and the measurements were performed on an automatic Synergy microplate reader (BioTek Instruments, Inc., Winooski, VT, USA) at a wavelength of 410 nm (0.25 mL sample per well). The fraction of consumed H_2_O_2_ was assessed using a calibration curve (0, 5, 10 and 20 mM of H_2_O_2_), and CAT activity was calculated as mM H_2_O_2_ per mL of blood per minute (mM/mL/min).

#### 2.4.4. Assessment of Pro-Oxidant/Antioxidant Ratio

The intensity of free radical processes in the blood was defined as pro-oxidant/antioxidant balance using the method of hydrogen peroxide-induced chemiluminescence (HPL) [31]. The total amount of light emitted in 3 min of the reaction, reflecting pro-oxidant/antioxidant ratio of the chemical products in the test sample, was analysed.

#### 2.4.5. Assessment of Oxidative Damage of Lipids

Lipid peroxidation (LPO) levels in the liver homogenates and blood plasma were evaluated using Stalnaya and Garishvili (1977) methodology [32]. This approach is based on the ability of malondialdehyde (MDA) to form a coloured complex containing one molecule of MDA and two molecules of 2-thiobarbituric acid (TBA).

#### 2.4.6. Estimation of Oxidative Modification of Proteins 

The level of oxidative protein modification (OPM) was determined using Levine et al. (1990) methodology as modified by Dubinina et al. (1995) [33,34]. This approach is based on the reaction of oxidized amino acid residues with 2,4-dinitrophenylhydrazine (DNPH), and formation of its derivatives. Using spectrophotometric analysis, the following parameters were determined: aldehyde phenylhydrazones (APH, at a wavelength of 274 nm), and ketone phenylhydrazones (KPH, at a wavelength of 370 nm). For a more complete picture of protein oxidation, both spontaneous and metal-catalysed OPM of plasma were studied.

### 2.5. Statistical Analysis

The data were expressed as the mean ± standard error of the mean (M ± m). Probability values with *p* < 0.05 were considered statistically significant. The distribution of parameters was estimated using the Shapiro–Wilk normality Test. Mann–Whitney U test and ANOVA test performed on Origin 7.5 software (Origin Pro 7.5 (OriginLab Corporation, Northampton, USA) were used to assess statistical significance of the differences between means.

## 3. Results

A single intravenous injection of Melphalan resulted in the instances of animal mortality, indicating severe toxicity of this alkylating agent. The mortality was more pronounced in the untreated group: three rats of the Melphalan Group (Group 2) died on the 6th day after Melphalan injection; two rats of the Melphalan + AC1 Group, and one rat of Melphalan + AC2 Group died. Animal body weight data before and after Melphalan injection are presented in Table 2.

On the 8th day following the intravenous Melphalan injection at a dose of 4 mg/kg, we observed a decrease in WBC by 55.6%, (neutrophils—by 50.0%, lymphocytes—by 62.7%), RBC—by 23.3%, haemoglobin level (HB)—by 19.4 % and platelets—by 43.1% (Table 3).

Compared to the Melphalan Group, in the animals of Melphalan + AC1 Group, WBC increased by 25.0%, while all other parameters increased insignificantly. In the Melphalan + AC2 Group, leukocyte count increased 1.5-fold (by 50.0%) in comparison to the Melphalan Group and by 20.0% (statistically significant) compared to the Melphalan + AC1 Group. Compared to the Melphalan Group, there was a 200% increase in neutrophils and 1.5 times of lymphocytes in the animals of Melphalan + AC2 Group. Platelet count in this group increased by 23.5% and haemoglobin level by 11.6% compared to the Melphalan Group.

ROS levels in the blood plasma of experimental animals are presented in Table 4. On the 8th day after Melphalan injection, there were only slight changes in the parameters, with a trend to elevated ROS production in the Melphalan Group (by 14.7% compared to the Control Group). ROS production levels in the animals of Groups 2 and 3 were between those in Groups 1 and 4, exceeding control value by 4.5–11.0%.

The comparatively low ROS increases can be explained by the fact that they were assessed on the 8th day after exposure to the alkylating agent. However, there is a trend towards normalized ROS production in the adsorbent Groups, in particular Melphalan + AC1 group.

Assessment of GSH levels in the liver tissues is presented in Table 5. Melphalan injection resulted in generally elevated GSH liver levels. Administering both oral adsorbents decreased the elevated levels of reduced glutathione, and, in the case of AC2, the difference was significant. Liver GSH levels of Melphalan treated animals that received adsorbents exceeded this value in the Control Group 1.09 times in the Melphalan + AC2 Group and 1.15 times in Melphalan + AC1 Group.

Thus, AC1 and AC2 administration to Melphalan exposed animals reduced GSH levels almost to the control values.

The data on CAT activity in the blood of experimental animals are presented in Table 6.

There were no significant changes in catalase activity in the blood of animals in all experimental groups. Animals of the Melphalan + AC1 Group had a slightly more pronounced stabilisation trend to the levels of the Control Group.

Even on the 8th day after Melphalan injection, we could detect elevated pro-oxidant/antioxidant ratio, by 21.2% (*p* < 0.05) in the Melphalan group compared to the Control Group (Table 7), indicating oxidative stress activation. Administration of AC2 resulted in a clear trend of reducing this value (by 16.4%, *p* < 0.1) compared to the Melphalan Group, while administration of AC1 resulted in a slight trend to decrease in the ratio.

Thus, Melphalan stimulated pro-oxidant processes in the animal body, inducing development of oxidative stress. AC2 was more effective reducing pro-oxidant/antioxidant ratio in the peripheral blood of rats.

An increase of MDA levels in the blood plasma after Melphalan injection was observed; however, it was insignificant compared to the Control Group (Table 8). 

Melphalan + AC2 Group animals had a significant reduction of MDA by 45.5% (*p* < 0.05), with this value being even lower than that of control animals. Such MDA reduction can be either due to the activation of defence antioxidant systems, or the direct effect of AC2 adsorbent, causing neutralization (decomposition) of MDA.

LPO processes in the liver of rats showed insignificant following the Melphalan injection (Table 9).

However, comparing the effect of both adsorbents on the level of MDA in the blood and liver shows that AC1 administration resulted in a more pronounced trend to reducing MDA levels in liver homogenates. At the same time, AC2 resulted in a more pronounced effect on the blood parameters (see Table 8).

LPO processes in the blood plasma intensified by the 8th day following the injection with a bifunctional alkylating agent Melphalan. Administering enterosorbents resulted in decreased MDA levels (by 16.4% for AC1, and 45.5% for AC2), falling, in some instances, even below the levels of the Control Group animals. Oral adsorbents had the opposite effect on blood and liver parameters, which is probably due to a different mechanism of their action. Thus, after the Melphalan injection, AC2 was more effective as a detoxification factor in the blood plasma, and AC1 in the liver.

Melphalan causes significant oxidative damage to plasma proteins. Protein oxidation can be induced either directly by ROS or indirectly by reactions with secondary by-products of oxidative stress (generated in lipid peroxidation, sugars oxidation, etc.). Spontaneous levels of APH and KPH exceeded the corresponding values of the Control Group, 3.8 times and 1.8 times, respectively (Table 10). Oral adsorbents significantly decreased the toxic effect of Melphalan: ketone derivatives levels were 1.7 and 1.6 times lower for AC1 and AC2 Groups respectively that of Melphalan Group. Both enterosorbent forms resulted in aldehyde derivative levels of OPM decreased approximately 1.3 times.

Induced APHD level was 1.25 times higher in the animals of Melphalan Group compared to the Control Group. At the same time, KPHD levels exceeded the corresponding values 2.5 times. This indicates that Melphalan binds with plasma proteins decreasing their antioxidant defence. Administration of oral adsorbent in both forms reduced the level of induced APHD: 1.4 and 1.6 times in the animals of Melphalan + AC1 and Melphalan + AC2 Groups, respectively.

However, these values were still significantly higher comparing to corresponding values of the Control Group. Induced KPHD levels were significantly lower at AC1 and AC2 administration after Melphalan injection. In the animals of Melphalan + AC1 Group it was reduced 1.6 times, and Melphalan + AC2 Group 1.4 times compared to the Melphalan Group animals. The effect of AC2 thus was more pronounced compared to AC1.

Data concerning oxidative modification of liver proteins are presented in Table 11.

Melphalan significantly increased levels of both spontaneous and induced OPM in the liver, which are the early markers of oxidative damage to macromolecules in the body. In the Melphalan Group, spontaneous and induced levels of aldehyde derivatives were 2.6 and 2.4 times higher and of ketone derivatives respectively 1.3 and 1.4 times higher compared to the Control Group.

Administration of oral adsorbents significantly suppressed Melphalan-induced oxidative modification of liver proteins. Spontaneous levels of APHD and KPDH were 1.9 and 1.2 times lower; and their respective induced levels 1.8 and 1.3 times lower respectively in of the animals of Melphalan + AC1 group compared to the corresponding values of Melphalan Group. AC2 administration resulted in 1.6 and 1.7 times decrease of spontaneous and induced levels of ADPH and 1.2 and 1.1 times decrease of spontaneous and induced KDPH levels compared to the animals of Melphalan Group.

## 4. Discussion

This study assesses the effect of Melphalan on haematological parameters and oxidative stress markers in female albino rats. It also evaluates a protective potential of two forms of oral carbon adsorbent: primary solid beads (AC1) and orally disintegrating micronized form (secondary granules, AC2). Cytotoxic drugs, including alkylating agents, are among the most effective agents for treating advanced ovarian and breast cancer, haematological malignancies such as leukaemia, multiple myeloma, and others; these drugs are also used for reduced-intensity conditioning regimens in allogeneic stem cell transplantation [4,35,36]. Myelosuppression is a dose-limiting factor for chemotherapy schemes [20,21,37] and thus control of such side-effects improves chemotherapeutic outcomes. The typical side effects of most anti-cancer drugs are ROS-dependent. Thus, if oxidative stress is alleviated, we can expect a decrease of the intensity of side effects. For further discussion of experimental results, it is necessary to characterise major features of Melphalan. Melphalan (L-phenylalanine mustard, phenylalanine mustard, L-PAM, or L-sarcolysin) is a potent cell cycle phase-nonspecific bifunctional alkylating agent, an analogue of sulphur mustard gas [38]. In this study, we observed undeniable signs of severe Melphalan-induced toxicity: 30% lethality (3 rats out of 10) in the Melphalan Group and significant loss of weight compared to the Control Group. Enteral sorption therapy decreased the rate of lethality: two rats died in Melphalan + AC1 Group and one rat in Melphalan + AC2 Group. This suggests a positive restorative effect of these oral adsorbents.

The main mechanisms of action of enterosorption, which explain their restorative effects, are the adsorption of different exogenous and endogenous toxins, among them the products of lipid and protein peroxidation [12,13,19,39,40]. It involves direct sorption of toxic products, exo- and endotoxins, pathogenic in the lumen of the gastrointestinal tract as well as conditionally pathogenic microorganisms and potential allergens. Direct contact of the enterosorbents with gastrointestinal mucosa structures affects their enzymatic composition, biological activity in the intestinal tissues and changes the functional state of the gastrointestinal tract. Sorbents can bind endotoxins from systemic bloodstream and internal media by direct diffusion from the blood and/or digestive juices. Additionally, adsorbents can indirectly stimulate the exchange and excretion of toxins by detoxifying organs, as well as binding and transporting biologically active substances, such as enzymes and bile acids to the surface of enterosorbent [12,13,14,15]. Long-term effects of enterosorption include hepatoprotective, nephroprotective, lipid-lowering, as well as alleviation in the severity allergies and other actions mediating detoxifying function of the organism [12,13,15,16,17,18].

In this study, AC2 administration promoted significant myeloprotective effect: 1.5-fold increase in leukocytes, 2-fold in neutrophils, 1.5-fold in lymphocytes, and 1.23-fold in platelet count compared to the animals in Melphalan Group, while AC1 administration resulted in a slight improvement trend in the haematological parameters.

The results of our experiment showed that Melphalan caused an increase in the values of pro-oxidant/antioxidant ratio, MDA levels, oxidative damage to plasma proteins and oxidative protein modification. This supports the results of previous studies, which demonstrated that cytotoxic effect of the drug is mediated through ROS-dependent cell death, in addition to alkylation of DNA and activation of the p53 pathway [36,37]. We observed only slight elevation of the other markers, which can be due to our experimental design: we assessed oxidative stress parameters on the 8th day after exposure to the alkylating agent, when the intensity of response has likely subsided. Furthermore, we performed the experiment on healthy rats and administered only one injection of Melphalan. These factors suggest that the high level of GSH in Melphalan Group animals was a compensation reaction of the organism. GSH is involved in scavenging ROS and is the first line of defence against oxidation.

It is important that the animals received enterosorbents two days before the Melphalan injection in addition to a dose they received after the injection. Usually, anti-cancer treatment consists of several courses of chemotherapy; as a result, the body can be already experiencing damage from previous treatment as well as because of the tumour. Thus, our results confirm that pre-treatment administration of enteral sorption therapy allows for decreasing the signs of systemic intoxication caused by the tumour and improves the detoxification capability of kidneys, liver, and other organs [13,20]. In particular, this demonstrated the results of liver function tests: on the 8th day after Melphalan injection, liver transaminases were still approximately 1.5 times higher in the animals Melphalan Group compared to Control Group. Sorption enteral therapy, especially with AC2 enterosorbent, resulted in less elevated markers of liver function, suggesting its improvement. These data are in press.

Both oral carbon adsorbents demonstrated capability to repair ROS-induced damage. Our findings support results of previous studies [12,13,18,20]. This comparative study of the effect of AC1 and AC2 enterosorbents administration on oxidative modification of plasma and liver proteins confirmed their capability to reduce indirectly the intensity of OPM, and consequently prevent the development of side effects of excessive and unrestrained oxidative stress induced by Melphalan. The differences in experimental outcomes data cannot be explained by the different adsorption capacity and pore size distribution of the AC1 and AC2 adsorbents, which are quite similar (Figure 4). We suggest that the difference is due to different kinetic parameters between AC1 (solid beads, Ø = 0.15–0.25 mm) and AC2 (one-micron size microparticles instantaneously dispersible in aqueous media). The primary beads and secondary granules (orally disintegrating micronized form) of carbon enterosorbent also have different pharmacokinetic and organoleptic properties. Solid beads retain their adsorptive activity at the lower part of the intestine, while orally disintegrating micronized forms undergo fast saturation starting in the oral cavity. This is important because of the frequent nausea and vomiting provoked by anti-cancer chemotherapy. Easier swallowing of the oral adsorbent (such the orally disintegrating micronized form) limits the vomiting response. Indeed, in this study, AC2 intake resulted in stronger response of the parameters such as MDA levels in the blood, spontaneous and induced levels of MDA in the liver tissues, and certain markers of oxidative modification of blood plasma proteins. This more pronounced response can be attributed to AC2 immediate and powerful action starting in the oral cavity. Different kinetic parameters of the two adsorbents were demonstrated in the methylene blue adsorption experiments, supporting the proposition of their different biological and therapeutic qualities. The difference in kinetic parameters likely also contributes to myeloprotective properties of AC2 in contrast to AC1.

An important remaining question is the impact of enterosorption on the antitumor effect of chemotherapy. ROS-dependent cell death is an intrinsic mechanism of action of alkylating agents [37,39]. Thus, oxidative stress regulation is an important factor in both neoplasia development and response to anticancer treatments. Our previous study [40] allows us to suggest that enterosorption offers protective outcomes with no negative impact on the cytostatic and anti-tumour effects. We analysed growth of Guerin’s carcinoma in rats with grafted tumours to show that administration of carbon enterosorbent, both alone and in combination with granulocyte colony-stimulating factor, did not affect antitumor effectiveness of the alkylating agent. A similar study using cisplatin demonstrated very similar results [22]. In the present study, we administered adsorbents to the rats at least three hours before and after the meal and excluded them for 24 h before and after Melphalan injection to avoid any possible impact to its pharmacokinetics. We took into account mean terminal half-life (t_1/2_ elim) of Melphalan, which is approximately 40–90 min depending on the dose of drug [41,42]. We chose such a wide interval between intravenous Melphalan administration and peroral carbon adsorbent administration to account for large variability in pharmacokinetic parameters among different individuals [42].

Our results support previous research which confirmed that modulation of oxidative stress markers alleviates toxicity of anti-neoplastic drugs [43,44,45].

## 5. Conclusions

Oxidative stress is a key factor contributing to side effects of anti-cancer drugs. Bone marrow suppression, neutropenia, and mucositis can force the patients and their doctors to limit the courses of dose-dense and dose-intense chemotherapy necessary for successful tumour treatment. Previous research showed promising results for the use of activated carbon materials as a part of Sorption Detoxification to diminish side effects of antineoplastic drugs. Results of this study closely align with our previous findings. Both forms of the newly developed oral adsorbent, AC1—primary solid beads of highly activated carbon from pyrolyzed proprietary phenolic resin and AC2—secondary orally disintegrating granules of micronized AC1, positively corrected a number of key oxidative stress markers on the animal model after a single Melphalan injection. For some parameters, such as ROS production in blood plasma, the use of enterosorbents demonstrated a slight positive improvement. We propose that this is because assessment of the markers took place on the 8th day after Melphalan injection, when the intensity of oxidative stress declined. However, both forms of oral carbon adsorbent significantly decreased oxidative modification of blood and liver proteins (especially the AC1 form), normalizing the level of reduced glutathione, pro-oxidant/antioxidant ratio and other parameters. The effect of primary beads was more pronounced in correcting oxidative stress markers. AC2 granules, on the other hand, were more effective in reducing pro-oxidant/antioxidant ratio and MDA levels. AC2 form also demonstrated a potent myeloprotective effect. The diversity of the effects of AC1 and AC2 on different parameters and markers can be explained by their different adsorption kinetic parameters, as indicated by the Methylene Blue adsorption tests, pharmacokinetic and organoleptic properties. Further studies should focus on the effects of both forms in combination.

There is a high demand for enterosorbents helping to alleviate anti-cancer chemotherapy side effects. Our results provide a solid foundation for further studies of oral carbon adsorbents and their introduction into clinical practice to decrease the adverse effects of anti-cancer treatment with cytotoxic drugs.

## Figures and Tables

**Table 1:** Kinetic data of Methylene Blue adsorption by primary beads (γ = 0.16 g/cm^3^) and corresponding microparticles of carbon enterosorbents (mg/g).

Time (hours)	Adsorption (mg/g of Carbon)	
	Primary Beads AC1	Secondary Beads AC2
1	589.0 ± 1.7	905.0 ± 1.2
2	640.0 ± 2.1	944.0 ± 3.3
3	681.0 ± 2.9	945.0 ± 4.1
24	818.0 ± 1.6	968.0 ± 3.4
72	820.0 ± 1.6	951.0 ± 3.4

AC—activated carbon.

**Table 2:** Body weight of the rats on the first and last days of the experiment.

Group	Body Weight (g)		
	Day 1	Day 10	Δ Days 1–10
Control, (*n* = 5)	228.0 ± 9.0	248.0 ± 9.0	20.2 ± 4.2
Melphalan, (*n* = 7)	220.0 ± 3.0	208.0 ± 8.0	−11.7 ± 6.5 ^a^
Melphalan + AC1, (*n* = 8)	226.0 ± 6.0	229.0 ± 7.0	2.6 ± 3.07 ^ab^
Melphalan + AC2, (*n* = 9)	226.0 ± 5.0	222.0 ± 8.0	−3.8 ± 4.6 ^a^

Notes. *p* < 0.05: 1. a—compared to the Control Group; 2. b—compared to Melphalan Group.

**Table 3:** Haematological parameters in rats on 10th day of the experiment (8th day after Melphalan injection).

Group	WBC, 10^9^/L	RBC, 10^12^/L	Hb, g/L	Platelets, 10^9^/L	Neutrophils, 10^9^/L	Lymphocytes, 10^9^/L
Control, (*n* = 5)	9.0 ± 0.8	6.0 ± 0.3	160.0 ± 3.0	577.0 ± 27.0	2.0 ± 0.2	6.7 ± 0.1
Melphalan, (*n* = 7)	4.0 ± 0.2 ^a^	4.6 ± 0.2 ^a^	129.0 ± 6.0 ^a^	328.0 ± 20.0 ^a^	1.0 ± 0.1 ^a^	2.5 ± 0.2 ^a^
Melphalan + AC1, (*n* = 8)	5.0 ± 0.3 ^ab^	4.8 ± 0.1 ^a^	140.0 ± 4.0	335.0 ± 15.0 ^a^	1.4 ± 0.2 ^a^	3.2 ± 0.4 ^a^
Melphalan + AC2, (*n* = 9)	6.0 ± 0.4 ^bc^	4.9 ± 0.2 ^a^	144.0 ± 4.0 ^ab^	405.0 ± 24.0 ^ab^	2.0 ± 0.5 ^b^	3.8 ± 0.4 ^ab^

Notes. *p* < 0.05: 1. a—compared to the Control Group; 2. b—compared to Melphalan Group; 3. c—compared to Melphalan + AC1 Group. White blood cell count (WBC), red blood cell count (RBC), haemoglobin level (Hb).

**Table 4:** The intensity of reactive oxygen species (ROS) formation in the blood of rats on the 8th day after Melphalan injection.

Groups	ROS Production, mM/L/min
Control, (*n* = 5)	1.67 ± 0.23
Melphalan, (*n* = 7)	1.94 ± 0.19
Melphalan + AC1, (*n* = 8)	1.76 ± 0.12
Melphalan + AC2, (*n* = 9)	1.87 ± 0.20

**Table 5:** Reduced glutathione levels in the liver on the 8th day after Melphalan injection.

Groups	Level of GSH, nm/mg
Control, (*n* = 5)	32.40 ± 5.91
Melphalan, (*n* = 7)	45.45 ± 3.45
Melphalan + AC1, (*n* = 8)	37.40 ± 1.38
Melphalan + AC2, (*n* = 9)	35.43 ± 2.81 ^a^

Notes. a—*p* < 0.05 compared to the Control Group. Reduced glutathione (GSH).

**Table 6:** Catalase (CAT) activity of in the blood of rats on the 8th day after Melphalan injection.

Groups	The Activity of CAT, mM/mL/min
Control, (*n* = 5)	15.63 ± 1.10
Melphalan, (*n* = 7)	13.73 ± 0.69
Melphalan + AC1, (*n* = 8)	14.68 ± 1.23
Melphalan + AC2, (*n* = 9)	13.97 ± 0.96

**Table 7:** Pro-oxidant/antioxidant ratio in the peripheral blood of rats on the 8th day after Melphalan injection.

Groups of Animals	Pro-Oxidant/Antioxidant Ratio, imp/180s
Control group, (*n* = 5)	89,838.40 ± 4539.35
Melphalan, (*n* = 7)	108,909.88 ± 4915.67 ^a^
Melphalan + AC1, (*n* = 8)	102,379.14 ± 7627.28
Melphalan + AC2, (*n* = 9)	91,095.60 ± 5191.38

Notes. a—*p* < 0.05 compared to the Control Group.

**Table 8:** Malondialdehyde (MDA) levels in the blood plasma of rats on the 8th day after Melphalan injection.

Groups	MDA Levels, μM/g Protein
Control, (*n* = 5)	67.32 ± 13.50
Melphalan, (*n* = 7)	83.06 ± 11.22
Melphalan + AC1, (*n* = 8)	83.36 ± 7.09
Melphalan + AC2, (*n* = 9)	45.25 ± 4.13 ^ab^

Notes. *p* < 0.05: a—compared to Melphalan Group; b—compared to Melphalan + AC1 Group.

**Table 9:** Spontaneous (mM/g protein) and induced (mM/g protein/30 min) levels of MDA in rat liver homogenates.

Groups	MDA Levels	
	mM/g Protein	mM/g Protein/30 min
Control, (*n* = 5)	0.15 ± 0.02	0.45 ± 0.04
Melphalan, (*n* = 7)	0.15 ± 0.01	0.47 ± 0.03
Melphalan + AC1, (*n* = 8)	0.13 ± 0.01	0.39 ± 0.01
Melphalan + AC2, (*n* = 9)	0.20 ± 0.03	0.45 ± 0.04

**Table 10:** Values of oxidative modification of blood plasma proteins.

Group	Spontaneous Level, μM/mg Protein		Induced Level, μM/mg Protein	
	APHD, M ± m	KPHD, M ± m	APHD, M ± m	KPHD, M ± m
Control, (*n* = 5)	3.66 ± 0.40	3.70 ± 0.18	8.00 ± 0.92	7.75 ± 0.78
Melphalan, (*n* = 7)	13.90 ± 0.45 ^a^	6.68 ± 0.55 ^a^	17.38 ± 0.77 ^a^	16.53 ± 1.14 ^a^
Melphalan + AC1, (*n* = 8)	10.88 ± 0.91 ^ab^	4.01 ± 0.33 ^b^	12.83 ± 1.18 ^ab^	10.33 ± 0.56 ^ab^
Melphalan + AC2, (*n* = 9)	10.55 ± 0.44 ^ab^	4.29 ± 0.29 ^b^	10.60 ± 0.35 ^ab^	12.02 ± 0.38 ^abc^

Notes. *p* < 0.05: 1. a—compared to the Control Group; 2. b—compared to Melphalan Group; 3. c—compared to Melphalan + AC1 Group. Aldehyde–dinitrophenylhydrazone derivatives (APHD), ketone dinitrophenylhydrazone derivatives (KPHD).

**Table 11:** Level of oxidative protein modification in liver homogenate of rats on the 8th day after Melphalan injection.

Group	Spontaneous Level, μM/mg Protein		Induced Level, μM/mg Protein	
	APDH, M ± m	KPDH, M ± m	APDH, M ± m	KPDH, M ± m
Control, (*n* = 5)	4.70 ± 0.52	5.30 ± 0.66	6.10 ± 0.42	23.76 ± 1.41
Melphalan, (*n* = 7)	12.17 ± 1.62 ^a^	7.11 ± 0.13 ^a^	14.92 ± 0.85 ^a^	33.52 ± 0.62 ^a^
Melphalan + AC1, (*n* = 8)	6.53 ± 1.14 ^b^	5.86 ± 0.20 ^b^	8.42 ± 0.89 ^ab^	25.77 ± 0.29 ^b^
Melphalan + AC2, (*n* = 9)	7.04 ± 0.80 ^ab^	6.09 ± 0.52	8.98 ± 0.44 ^ab^	29.60 ± 0.59 ^abc^

Notes. *p* < 0.05: 1. a—compared to Control Group; 2. b—compared to Melphalan Group; 3. c—compared to Melphalan + AC1 Group.
